# Tenacity of Animal Disease Viruses on Wood Surfaces Relevant to Animal Husbandry

**DOI:** 10.3390/v16050789

**Published:** 2024-05-15

**Authors:** Martin J. Oettler, Franz J. Conraths, Uwe Roesler, Sven Reiche, Timo Homeier-Bachmann, Nicolai Denzin

**Affiliations:** 1 Institute of Epidemiology, Friedrich-Loeffler-Institut, Federal Research Institute for Animal Health, 17493 Greifswald, Germany; franz.conraths@fli.de (F.J.C.); timo.homeier@fli.de (T.H.-B.); nicolai.denzin@fli.de (N.D.); 2 Institute for Animal Hygiene and Environmental Health, Freie Universität Berlin, 14163 Berlin, Germany; uwe.roesler@fu-berlin.de; 3 Department of Experimental Animal Facilities and Biorisk Management, Friedrich-Loeffler-Institut, Federal Research Institute for Animal Health, 17493 Greifswald, Germany; sven.reiche@fli.de

**Keywords:** hygiene, farm building, wood, Enterovirus E, Newcastle disease virus

## Abstract

The aim of this study was to analyse the hygienic suitability of wood often used in animal husbandry. To this end, the inactivation of viruses (Enterovirus E as a surrogate for non-enveloped viruses and Newcastle disease virus as a surrogate for enveloped viruses) on germ carriers consisting of various types of wood was studied over an extended period to assess the biosafety of wood as an agricultural building material. The study was designed to assess the intrinsic biocidal activity of the wood itself, without the use of a disinfectant. The laboratory tests were based on German test guidelines and current European standards. Five different types of wood germ carriers, i.e., spruce (*Picea abies*), pine (*Pinus sylvestris*), poplar (*Populus* sp.), beech (*Fagus sylvatica*) and Douglas fir (*Pseudotsuga menziesii*), as well as stainless-steel carriers, were inoculated with enveloped and non-enveloped viruses and stored for up to four months, and the remaining infectivity of the viruses was continuously assessed. The results showed that intact, finely sawn timber with a low depth of roughness had an inactivating effect on the viruses up to 7.5 decadal logarithmic levels. For the non-enveloped virus, inactivation was fastest on Douglas fir wood, with the target reduction for effective inactivation (reduction by factor 4.0 log_10_) being achieved after two weeks, and for the enveloped virus on pine wood, it was already achieved from the day of drying. The hygienic effects of the wood carriers may be due to their hygroscopic properties and wood constituents. These effects offer potential for further investigation, including tests with other wood species rich in extractives.

## 1. Introduction

Wood or timber represent predominant building materials in many farm buildings in various countries including Germany [1,2]. In addition, farmers are encouraged to use timber in agriculture as a renewable resource [3]. Yet, wood is believed to be difficult to sanitise in the context of animal disease control [4], while studies from the food sector have presented contrary evidence, attributing good hygienic properties to wooden surfaces [5,6]. One of the reasons for these positive effects may be the presence of wood ingredients [7].

Repopulation of an animal building must not take place before sufficient time has elapsed following the completion of cleaning and disinfection in the context of animal disease control. In the European Union, this is regulated by law [8]. An additional safety factor is meant to reduce any risk of remaining infectious animal pathogens even further. However, infectious material may remain unreached in niches and cracks of wooden constructions despite thorough cleaning and disinfection. It is therefore of interest to assess how long typical non-enveloped and enveloped viruses can remain infectious on wooden surfaces that have not been exposed to disinfectants. Thus, the risk that such pockets of residual infectious agents serve as a source of infection for restocked animals needs to be assessed, taking the legally defined vacancy periods into consideration.

There are no standardised test protocols or guidelines for long-term testing of virus inactivation on surfaces. However, there are guidelines for determining pathogen resistance to disinfectants [9,10,11]. These guidelines refer to enveloped as well as non-enveloped viruses if virucidal efficacy is to be assessed, as resistance is affected by the presence of a lipid envelope [12]. In general, enveloped viruses are considered to be less resistant than non-enveloped viruses [13,14,15,16].

The use of wood as a conventional building material in animal husbandry holds the potential to rely on a renewable resource, thus fostering a more sustainable economy, promoting climate protection and enhancing regional value chains. Nevertheless, these considerations should not interfere with the aspects of animal hygiene and animal disease control.

We thus set out to assess how long typical enveloped (i.e., Newcastle disease virus) and non-enveloped (i.e., Enterovirus E) viruses can remain infectious on wooden surfaces that have not been exposed to disinfectants.

## 2. Materials and Methods

### 2.1. Cells and Viruses

Cell culture and virus propagation procedures adhered to the prevailing European standards [10].

In brief, Leghorn male chicken hepatocellular carcinoma (LMH, CCLV-0417) cells and Madin–Darby bovine kidney (MDBK, CCLV-0261) cells were cultured in their respective cell culture media—ZB9h (Appendix A, Table A1) for LMH and ZB5 (Appendix A, Table A2) for MDBK; each were supplemented with 10% foetal calf serum (FCS). Cell cultures were incubated at 37 °C and 5% CO_2_. Both cell lines were obtained from the Bio Bank (Department of Experimental Animal Facilities and Biorisk Management) of the Friedrich-Loeffler-Institut, Federal Institute for Animal Health, Germany.

The standard test viruses listed in the methods outlined by the German Veterinary Medical Society (Deutsche Veterinärmedizinische Gesellschaft, DVG) for testing chemical disinfectants in animal husbandry [9] were employed as surrogates for relevant animal disease viruses. They represent prototypes for viruses that can cause important animal diseases and serve as model viruses in the testing of the required disinfectant concentrations used to control these diseases [17].

Newcastle disease virus (NDV, strain Montana) was used as a model for enveloped viruses and Enterovirus E (EV-E, strain LCR 4) as a model for non-enveloped viruses.

LMH cells were used for propagation and as a detection system for NDV, while MDBK cells were used for EV-E. Following virus propagation, the viruses were aliquoted into cryotubes and stored as a virus test suspension at −80 °C. Both virus strains were supplied by the Institute for Animal Hygiene and Environmental Health of the Freie Universität Berlin, Germany.

### 2.2. Germ Carriers

**Figure 1:**
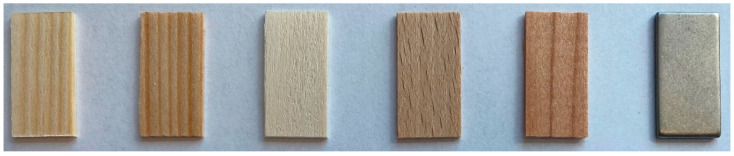
Germ carriers (from left to right: spruce, pine, poplar, beech, Douglas fir, stainless steel).

In laboratory tests, germ carriers made of both porous and non-porous materials were used (Figure 1). The porous materials comprised real wood discs made from five different types of wood, while stainless-steel sheets were chosen as the non-porous material.

Among the wooden carriers used were discs of spruce (*Picea abies*) and pine (*Pinus sylvestris*), as these are two classic German woods used in construction. Poplar (*Populus* sp.) discs were also analysed, as this is the German [9] and European [18] standard for tenacity testing on porous materials in animal husbandry. Beech (*Fagus sylvatica*) and Douglas fir (*Pseudotsuga menziesii*) discs were included in the test as representatives of modern construction timber. The wooden germ carriers were cut from dried boards and sawn to a size of 20 mm × 10 mm and a thickness of 1 mm before testing. The surface was classified as fine-sawn and thus had a uniform surface with a low roughness depth [19]. The wood discs were prepared and kindly provided by the Chair of Wood Science (School of Life Sciences) of the Technical University of Munich, Germany.

A steel carrier was analysed as this is the European standard test carrier for virucidal tenacity tests on non-porous surfaces for the veterinary sector [11]. The thin sheets were 20 mm × 10 mm in size and 1.25 mm thick. The material used was 1.4301 stainless steel [20]. This steel grade provides good corrosion resistance to environmental influences due to the chromium oxide protective layer [20]. The surface quality was 2B on both sides [21]. This quality is characterised by good smoothness, flatness and corrosion resistance [21]. The steel plates were ordered according to the specifications mentioned above and purchased from ottim Metall GmbH, Berlin, Germany.

### 2.3. Performing the Tenacity Tests

The laboratory tenacity tests were conducted following the German DVG test guideline [9] in combination with current European standards [11,18]. For each long-term test, 27 carriers per wood species (three per time point) and 54 steel carriers (two different tests with 27 carriers each) were used. The evaluation was carried out at nine different test time points. At each time point, 3 of the 27 carriers were withdrawn from the test and analysed for any remaining infectious virus.

Prior to testing, the wood germ carriers were sterilised with autoclaving (121 °C, 2.8 MPa for 1.5 h) and then dried. The steel plates were first placed in a surfactant solution (5% Decon 90^®^, Decon Laboratories Ltd., Hove, UK) for one hour, then rinsed with demineralised water, degreased with 2-propanol for 15 min and rinsed again. The subsequent autoclaving and drying process was identical to that applied to the wooden germ carriers.

For inoculation, the virus test suspension from the virus propagation was mixed with 3 g/L bovine serum albumin (Sigma-Aldrich Chemie GmbH, Steinheim, Germany) as a potentially interfering substance. Each germ carrier surface was inoculated with 100 µL of this mixture. Subsequent drying of the inoculated wood and half of the steel (hereafter referred to as “steel”) carriers was carried out using a desiccator with silica gel. The sealed desiccator was placed in a climate chamber at 10 °C for one day. After drying, the germ carriers were transferred into the wells of lidded 6-well plates for long-term storage in the climate chamber at 10 °C.

In order to avoid potential condensation of humidity on the steel carriers during incubation, the remaining second half of the steel germ carriers (hereafter referred to as “steel*”) were treated differently. Here, the inoculated steel carriers were completely dried in a silica gel desiccator under vacuum (approximately 80 kPa negative pressure) at room temperature. These carriers were then also transferred to the wells of lidded 6-well plates. The gaps and unused wells of the microtitre plate were filled with silica gel. The plates were then airtightly sealed with a sealing foil and self-adhesive aluminium foil. Long-term storage was performed together with the unsealed 6-well plates in a climate chamber at 10 °C.

Titre evaluation was performed in triplicate at nine time points. Accordingly, three carriers per carrier type were withdrawn from the test and evaluated at each time point. The total duration and interval of the long-term observations for each test virus were determined by preliminary tests. The experiment lasted 112 days for EV-E and 35 days for NDV, respectively.

The first evaluation was performed when the carriers were transferred from the desiccator to the 6-well plates or on the day after the sealing of the steel carriers (steel*), respectively. This day was defined as the day of drying or day “zero” of the experiments. For EV-E, further evaluation was performed at 7-day intervals after drying or at later evaluation times at 14-day or 28-day intervals. For NDV, the evaluation was carried out at 5-day intervals after the day of drying. In addition, germ carriers were evaluated already three days after drying to provide a more accurate record of potential titre losses at the beginning of the test period.

**Figure 2:**
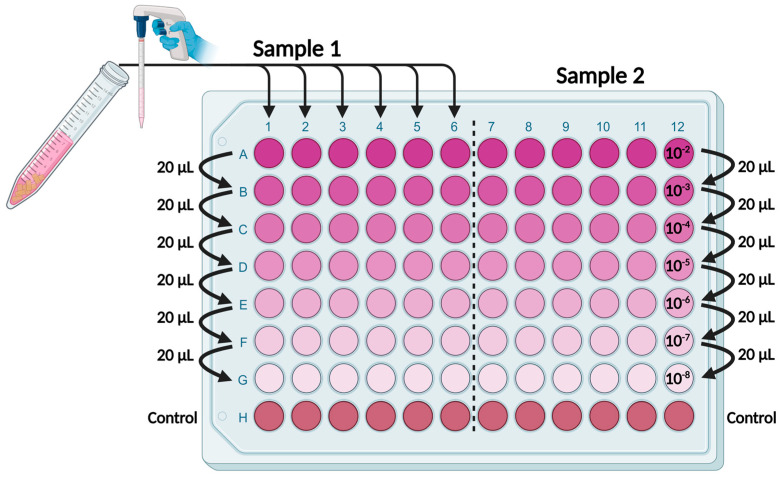
Titration scheme on a 96-well plate, created with Biorender.com.

At each time point, the respective germ carriers were withdrawn from incubation and transferred into 9.9 mL of the respective cell culture medium (containing 2% FCS). The wooden germ carriers were cut into eight pieces using sterile scissors, while the steel carriers were left intact. To desorb the viruses, the suspension was vortexed, treated in an ultrasonic bath filled with ice water for 10 min and then centrifuged at 1600× *g_N_* for 15 min. Thus, the initial virus titre was diluted by factor 10^−2^. Titration of the supernatant was carried out in a 96-well plate (Figure 2).

The wells of rows B-H were prefilled with 180 µL of the respective cell culture medium (containing 2% FCS). At least 120 µL of the virus sample suspension was transferred to row A; then, 20 µL was pipetted into the next dilution row and mixed, and the process was repeated up to row G. Row H served as a negative control without the addition of virus dilutions. For the incubation of a titration sample, 100 µL was transferred from each well to a 96-well cell culture plate and incubated at 37 °C and 5% CO_2_. In addition, to detect low virus load, 1 mL was directly transferred from the falcon tube six times to the wells of a 12-well cell culture plate and incubated under the same conditions.

The analysis was conducted based on virus-specific cytopathic effects (CPEs). Virus titres were calculated using the Spearman–Kärber formula (Appendix A, Equation (A1)) [22,23]. In order to calculate the virus reduction, the test titre was subtracted from the initial titre (liquid virus test suspension with loading substance directly in a 9.9 mL cell culture medium containing 2% FCS).

For each test time point in the long-term observations, the average virus reduction in the triple test was calculated and evaluated on the basis of logarithmic values. The time point when no more virus could be quantitatively detected and the time point when an effective inactivation (reduction in virus concentration of at least four decadal logarithmic levels [9]) had been achieved were checked.

### 2.4. Statistics

The software R version 4.3. for Windows [24] was used for significance testing. Considering the limited number of data points, it was conservatively assumed that the values were not normally distributed. Thus, the non-parametric Kruskal–Wallis test was applied to compare log titre reductions, and a *p*-value of 0.05 was set as the significance level for differences to be detected.

## 3. Results

### 3.1. Visualisation of the Cytopathic Effect

**Figure 3:**
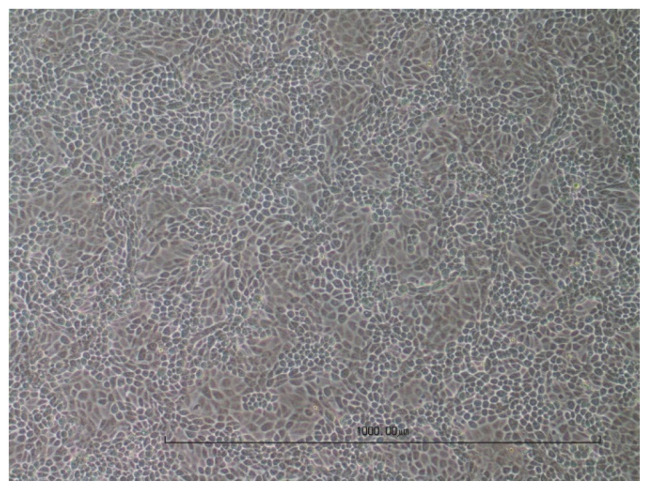
Native MDBK cells.

**Figure 4:**
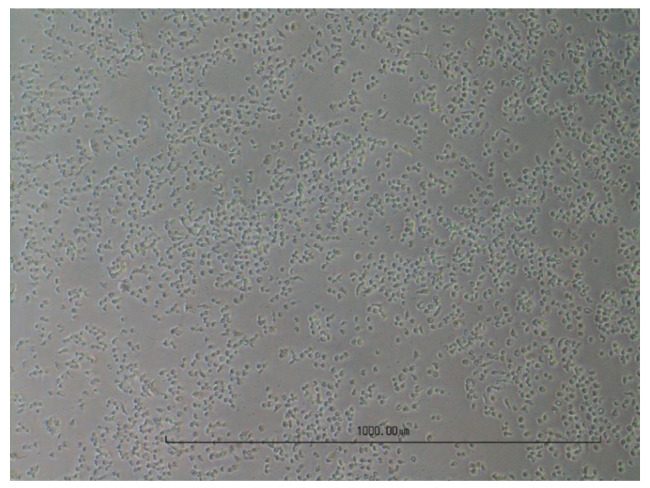
Fully lysed MDBK cells after infection with EV-E.

**Figure 5:**
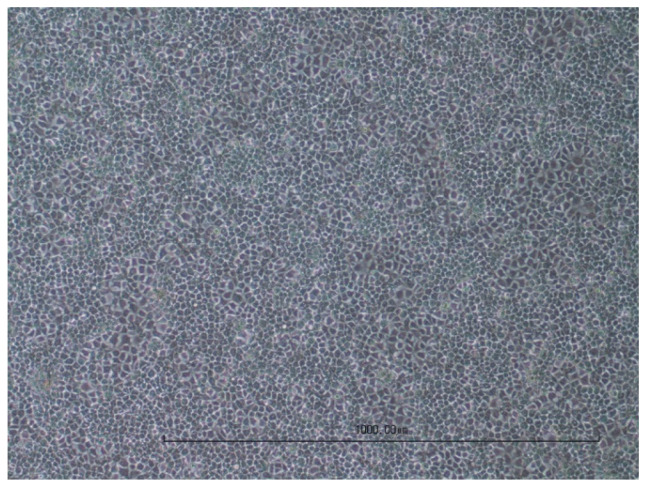
Native LMH cells.

**Figure 6:**
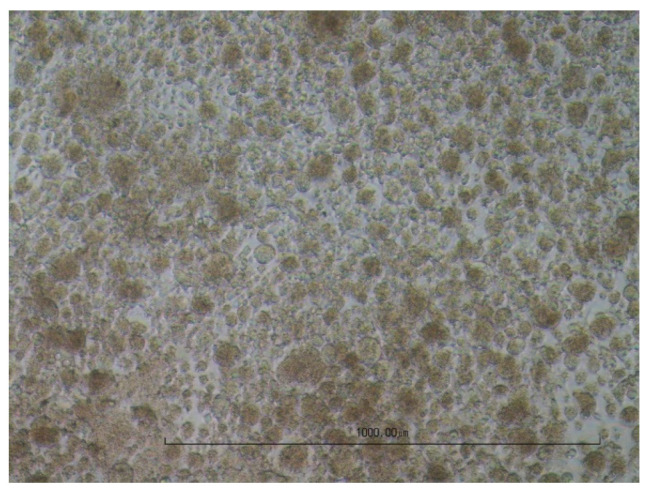
Fully lysed LMH cells after infection with NDV; giant cell formation.

The cell cultures were digitally photographed to visualise the cytopathic effect. As examples, native and fully lysed cell layers with the virus-specific CPE for MDBK (Figure 3 and Figure 4) and for LMH (Figure 5 and Figure 6) are shown. Cell cultures in a 12-well plate were used for the images at 10× magnification.

### 3.2. Steel Carriers

Due to the high humidity in the climate chamber, condensation formed on the surface of the steel sample (steel) after the initial drying process with the consequence that steel carriers never dried over the entire period. However, for the alternative sample (steel*), condensation was prevented by sealing the panels with silica gel. This adaptation allowed us to also obtain test results for dried steel.

### 3.3. Long-Term Observations of the Tenacity of EV-E

**Figure 7:**
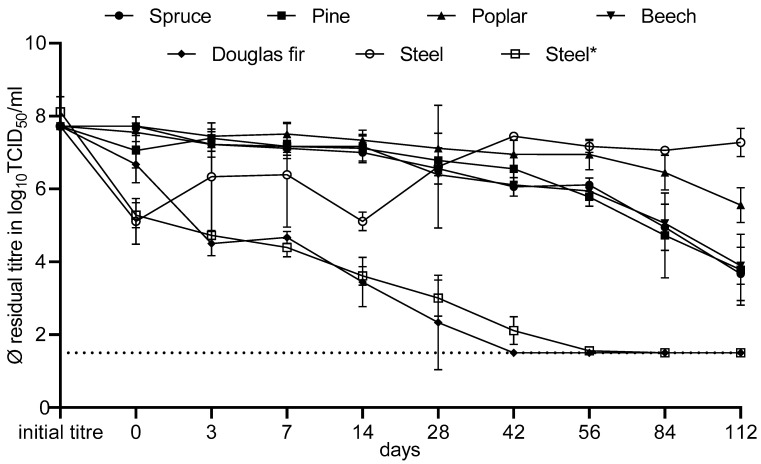
Average titre reduction of EV-E on different surfaces with standard deviations.

In the long-term observations of EV-E persistence on the different surfaces, a statistically significant difference (*p* < 0.05) in titre reduction among the various wood species was observed from day 28 onwards (Figure 7). 

Douglas fir wood showed the best overall intrinsic biocidal efficacy. After two weeks, the average titre reduction on the latter wood was 4.28 log_10_TCID_50_/mL, thus achieving the target reduction as defined for an effective inactivation (reduction by factor 4.0 log_10_). After six weeks and until the end of the test, no virus could be detected on the Douglas fir wood, and the reduction was ≥6.22 log_10_TCID_50_/mL based on the initial titre and the limit of detection. The second-best result was achieved with the carrier steel*. Again, a result (average reduction of 4.50 log_10_TCID_50_/mL) corresponding to successful inactivation was achieved after two weeks. After 12 weeks, no virus was detectable and the reduction was ≥6.61 log_10_TCID_50_/mL. Spruce, pine and beech performed less effectively. Spruce wood led to successful inactivation after approximately four months of incubation, while pine and beech only came close to a four-log-level reduction. After week 16, the titre reduction on spruce wood was 4.06 log_10_TCID_50_/mL, on pine wood 3.94 log_10_TCID_50_/mL and on beech wood 3.83 log_10_TCID_50_/mL. The reduction in poplar wood was limited to 2.17 log_10_TCID_50_/mL only after week 16. The lowest inactivation efficacy at the end of the trial was found for the steel carrier. The EV-E titre reduction after 16 weeks was only 0.44 log_10_TCID_50_/mL. However, it should be noted that during the course of the trial (especially at the beginning), the titre reduction was sometimes much higher.

### 3.4. Long-Term Observations of the Tenacity of NDV

**Figure 8:**
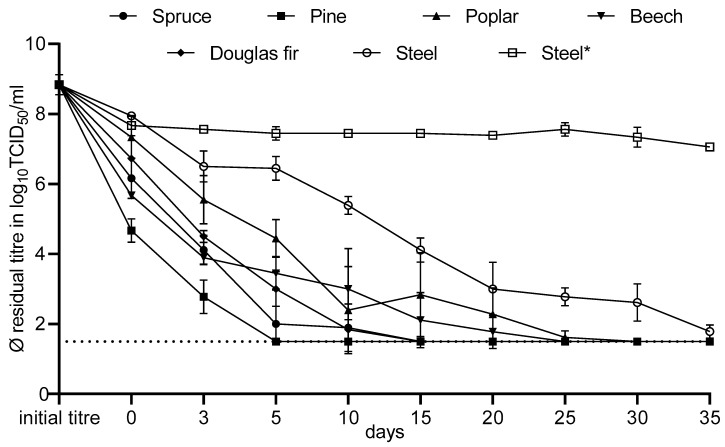
Average titre reduction of NDV on different surfaces with standard deviations.

In the long-term observations of NDV persistence on the different surfaces, starting from the day of drying (day zero), a statistically significant difference (*p* < 0.05) in titre reductions among the wood species could be detected (Figure 8).

Overall, pine wood showed the best intrinsic biocidal activity. Already on the day of drying, the titre reduction was 4.17 log_10_TCID_50_/mL. This corresponds to an effective inactivation of at least four decadal log levels. After five days, the infectious virus could no longer be detected on the pine germ carriers, and the titre reduction was ≥7.33 log_10_TCID_50_/mL, based on the initial titre and the detection limit. This result was sustained, as no virus was detected on the pine germ carriers in subsequent analyses up to day 35. The next best results were obtained with the other two conifers, spruce and Douglas fir. The results corresponding to successful inactivation were observed from day three onward, with a reduction of ≥4.72 log_10_TCID_50_/mL and 4.33 log_10_TCID_50_/mL on spruce wood and Douglas fir wood, respectively. From day 15 on, no residual virus could be detected anymore on both wood species, and the maximum titre reduction (≥7.33 log_10_TCID_50_/mL) was achieved. Next in performance were the two deciduous woods, beech and poplar. Effective inactivation was observed on beech from day three on with a reduction of ≥4.94 log_10_TCID_50_/mL and on poplar from day five on with a reduction of 4.39 log_10_TCID_50_/mL. NDV was no longer detectable on beech wood from day 25 and on poplar wood from day 30 on. On the contrary, with the steel carrier (steel), only a reduction equivalent to successful inactivation was achieved on day 15 (4.72 log_10_TCID_50_/mL) and onward. At the end of the test period on day 35, some residual infectious virus could still be detected. The titre reduction at this time was ≥7.06 log_10_TCID_50_/mL. The lowest inactivation efficacy by far was achieved with the alternative steel carrier (steel*). The maximum virus reduction over the entire test period was only 1.78 log_10_TCID_50_/mL on day 35. It should be noted that the reduction on day zero was already 1.17 log_10_TCID_50_/mL, indicating that there was only a slight reduction in titre over the following 35 days.

### 3.5. Detailed Results

The full results can be found in the Supplementary Materials (Tables S1 and S2).

## 4. Discussion

The results of the long-term observation regarding the decline of virus infectivity on wood and steel carriers generally indicated that EV-E had a considerably higher tenacity than NDV on the wood germ carriers. This result is consistent with the fact that non-enveloped viruses are generally more resistant to environmental influences than enveloped viruses [13,14,15,16]. A comparison of the results of the wooden and steel carriers used showed that in most cases, the wood had a better virus reduction effect than the steel. This result is consistent with the large long-term inactivation effect of wooden stable components described by Thiel [25], compared with the rather poor effect of metal surfaces. The different results for the individual woods may be linked to properties that depend on the wood species (Table 1). Concerning the extract content, in the initial autoclaving process of the presented test approaches, many extractives are lost due to thermal treatment [26]. Thus, under practical conditions, extractives could have an even greater effect on virus reduction. The standardised laboratory conditions in the climate chamber with a high relative humidity of on average 89% are also only partially comparable to variable real-life conditions in a stable.

### 4.1. Long-Term Observations of the Tenacity of EV-E

For the non-enveloped Enterovirus E, the high resistance to environmental influences, also shown by Olszewska et al. [27], Biermann et al. [28] and Steiger [29], was confirmed. An exception among the woods was Douglas fir, which showed a clear virucidal effect on EV-E already from the third day of the long-term evaluation. This effect may be related to the low pH of Douglas fir wood (Table 1). According to Rheinbaben and Wolff [30], EV-E is stable in the pH range of 4 to 8. In terms of other potentially active properties of wood components (e.g., tannins or resins) and the hygroscopic effect of the porous surface, Douglas fir is not unique among the woods used. However, in contrast to Douglas fir, the other wood surfaces showed a much slower decrease in virus titre. The similarly effective and continuous reduction in titre over time on the steel* carrier can be explained by the technically enforced drying of the virus throughout incubation, which was implemented to avoid rehydration with condensation water in the climate chamber, thus simulating practical conditions (with good ventilation) for the steel surface. The higher reduction (at the limit of detection) on the steel carriers as compared to Douglas fir was due to the fact that the steel* samples were prepared in a separate assay and therefore had a somewhat different initial titre. Without these specific measures of drying, the inoculum on the alternative carrier (steel) underwent re-suspension with the formation of a persistent liquid layer on the surface after the initial drying. Titre reduction on this carrier was very low even after about four months. This observation matches the experience of Rheinbaben and Wolff [30], who reported that EV-E remains infectious in an aqueous environment at 4 °C for a period of two years. Olszewska et al. [27] also demonstrated that EV-E showed almost no loss in titre after 135 days in an aqueous environment at 4 °C. The distinct variation in virus titre on the steel carrier (steel) between and within test time points in the early phase of the long-term evaluation (Figure 7) may be attributed to variation in the process of the initial drying step, as it can be ruled out that the viruses were replicating on the germ carriers. Specifically concerning the steel carriers, drying was faster and more effective for those carriers situated more marginally than centrally on a plate inside the desiccator.

According to Rhee et al. [31], EV-E is a surrogate virus for foot-and-mouth disease (FMD) virus, so the vacancy times after final disinfection legally prescribed for the latter animal disease can be roughly compared with the results of the tenacity tests in order to evaluate the biological plausibility of the legal requirements. According to the German FMD regulations, stables can only be restocked three months after final disinfection; earlier restocking is only permitted in conjunction with clinical examinations of the restocked livestock [32]. After a period of approximately three months, the average virus reduction on spruce, pine and beech construction wood was already 2.67–3.0 log_10_TCID_50_/mL without the use of any disinfectant. Douglas fir and dry steel (steel*) performed even better with probably, considering the quantitative detection limit, complete virus inactivation after the specified time period. Thus, it can be assumed that the legal requirements for FMD ensure a negligible risk of infection for any restocked animals through a combination of cleaning and disinfection, as well as subsequent vacancy, in stables consisting of the respective building materials.

### 4.2. Long-Term Observations of the Tenacity of NDV

Regarding the enveloped Newcastle disease virus, the long-term tests on coniferous wood, especially pine, lead to virus inactivation in a shorter time than on deciduous wood. This difference may be explained by the higher content of wood constituents (Table 1). These high extract contents, especially for pine, also correlate with the much higher number of resin ducts and their greater width [33,34]. As already described by Thiel [25], all woods analysed showed a faster reduction in titre than the two different steel carrier experiments, which may be explained by hygroscopic effects and the wood constituents contained. On steel without enforced drying (steel), virus reduction was slower and the residual titre at the end of the test period was just above the detection limit. This is consistent with the description by Kaaden [35] that NDV remains infectious for 30 to 35 days in infected stables, depending on the ambient temperature. Frölich et al. [36] also reported a similar period of 18 to 36 days for the survival of NDV in cages. Concerning the steel carrier with enforced drying (steel*), only very limited virus reduction was observed over the entire test period as compared to all other germ carrier types. As described by Steiger [29], NDV can maintain its infectivity over a long period of time in a dried and thus protected state. Kaaden [35] also mentioned the phenomenon that the infectivity of the dried virus can be maintained for years.

According to German animal disease regulations, the restocking ban after an outbreak of Newcastle disease is set at 30 days after completion and approval of disinfection [37]. Our results showed that even without prior disinfection, there was a high probability (the restriction here is due to the quantitative detection limit) that no infectious virus was present anymore on any of the construction timber tested after 30 days, while residual infectivity was still detectable on the moist steel surface (steel) and a very high residual titre was still detectable on the dried steel surface (steel*). Therefore, it can be assumed that the legal requirements for Newcastle disease ensure a negligible risk of infection for restocked animals through a combination of cleaning and disinfection followed by vacancy of premises. Particular attention should be paid to cleaning and disinfection in areas of the buildings with built-in steel, where NDV, e.g., due to good ventilation, may persist infectious in a dried and therefore protected state.

## 5. Conclusions

The hygienic properties of traditional and anticipated future construction woods were evaluated with respect to the tenacity of surrogates of relevant animal disease viruses. The results of the long-term observations on wood surfaces indicate that legally established vacancy periods of farm buildings after cleaning and disinfection in the course of outbreak management markedly contribute to the reduction of the risk of pathogen persistence and thus infection of restocked domestic animals. Virus inactivation on the fine-sawn timbers tested may be related to hygroscopic properties and particular wood constituents. These effects offer potential for further investigation, including tests with other wood species rich in extractives.

## Figures and Tables

**Table 1:** Wood characteristics according to Grosser and Teetz [7].

	Spruce	Pine	Poplar	Beech	Douglas Fir
Ø bulk density ^1^	0.43 g/cm^3^	0.49 g/cm^3^	0.43 g/cm^3^	0.68 g/cm^3^	0.47 g/cm^3^
Ø bulk density ^2^	0.47 g/cm^3^	0.52 g/cm^3^	0.47 g/cm^3^	0.72 g/cm^3^	0.51 g/cm^3^
pH value	5.0	5.1	5.8	5.1–5.4	3.3–4.2
Extract content	2.3%	9.0%	N/A	1.5%	6.0%

^1^ at 0% wood moisture; ^2^ at 15% wood moisture.

## Data Availability

The original contributions presented in the study are included in the article and Supplementary Materials; further inquiries can be directed to the corresponding author.

## Supplementary Materials

The following supporting information can be downloaded at: https://www.mdpi.com/article/10.3390/v16050789/s1; Table S1. Residual titre in log_10_TCID_50_/mL of EV-E on germ carriers over time and with three replicates each., Table S2. Residual titre in log_10_TCID_50_/mL of NDV on germ carriers over time and with three replicates each.

## Appendix A

**Table A1:** Recipe for cell culture medium ZB9h.

Component	Per Litre
DMEM	9.9 g
NaHCO_3_	860 mg
pH 7.2	

**Table A2:** Recipe for cell culture medium ZB5.

Component	Per Litre
MEM Eagle (Hanks’ Salts)	5.32 g
MEM (Earles’ Salts)	4.76 g
NaHCO_3_	1.25 g
Non-essential amino acids (NEA, 100×)	10 mL
Na-pyruvate	120 mg
pH 7.2	

Equation (A1) Spearman–Kärber formula [22,23].

m = x_k_ + d/2 − d Σ p_i_ (A1)

m: The negative decadic logarithm of the titre based on the test volume in the wells, given in log TCID_50_/volume.
x_k_: The logarithm value of the lowest dilution level at which all wells of a dilution series have a CPE.
d: The logarithm value of the dilution factor.
p_i_: The observed reaction rate (proportion of wells with CPE in a row).
Σ p_i_: The sum of the reaction rate from x_k_ to the highest dilution level.
