# Supplementary material for: Tenacity of Animal Disease Viruses on Wood Surfaces Relevant to Animal Husbandry
Source: Viruses. 2024 May 15;16(5):789. doi: 10.3390/v16050789 (PMC11125591; doi:10.3390/v16050789)
Supplement: Supplementary file 1 [file viruses-16-00789-s001.zip › viruses-3011564-supplementary.pdf]

Table S1. Residual titre in $log_{10}TCID_{50}$/mL of EV-E on germ carriers over time and with three replicates each.

	Day 0	Day 3	Day 7	Day 14	Day 28	Day 42	Day 56	Day 84	Day 112
	8.00	7.33	7.00	7.00	6.67	5.83	6.33	5.67	4.17
**Spruce**	7.67	7.33	7.00	7.00	6.67	6.00	6.00	4.67	4.00
	7.50	7.00	7.33	7.00	6.33	6.33	6.00	4.50	2.83
	6.50	7.17	6.83	6.83	6.50	6.33	5.83	3.50	4.50
**Pine**	7.33	7.67	7.17	7.50	6.67	6.33	5.50	5.83	2.67
	7.33	7.33	7.50	7.00	7.17	7.00	6.00	4.83	4.17
	7.67	7.50	7.67	7.50	7.50	6.50	7.00	6.17	5.83
**Poplar**	7.83	7.50	7.17	7.17	6.67	7.17	6.50	7.00	5.83
	7.67	7.33	7.67	7.33	7.17	7.17	7.33	6.17	5.00
	7.83	7.50	7.17	7.00	6.67	6.17	5.83	5.17	4.33
**Beech**	7.33	7.33	7.00	7.67	6.17	6.17	6.00	5.00	3.33
	7.50	6.83	7.33	6.83	6.33	6.00	6.00	5.00	4.00
	6.17	4.50	4.83	≤ 3.33	3.83	≤ 1.50	≤ 1.50	≤ 1.50	≤ 1.50
**Douglas fir**	7.17	4.17	4.67	4.17	≤ 1.50	≤ 1.50	≤ 1.50	≤ 1.50	≤ 1.50
	6.67	4.83	4.50	≤ 2.83	≤ 1.67	≤ 1.50	≤ 1.50	≤ 1.50	≤ 1.50
	5.83	7.50	7.67	5.33	7.67	7.50	7.33	7.17	7.50
**Steel**	4.67	4.67	4.83	5.17	7.50	7.33	7.00	7.00	6.83
	4.83	6.83	6.67	4.83	4.67	7.50	7.17	7.00	7.50
	5.67	4.67	4.33	≤ 3.33	2.50	≤ 1.67	≤ 1.67	≤ 1.50	≤ 1.50
**Steel***	5.00	4.83	4.67	3.83	3.50	≤ 2.33	≤ 1.50	≤ 1.50	≤ 1.50
	5.17	4.67	4.17	3.67	3.00	≤ 2.33	≤ 1.50	≤ 1.50	≤ 1.50

* completely dried in a desiccator and then stored airtightly sealed

**Initial titre**[1]	7.67	7.67	7.88	**Initial titre**[2]	8.17	7.67	8.50

[1] Initial titre in suspension on the day of infection; virus used for all wood carriers and steel
[2] Initial titre in suspension on the day of infection; virus used for steel*

Table S2. Residual titre in $log_{10}TCID_{50}/mL$ of NDV on germ carriers over time and with three replicates each.

	**Day 0**	**Day 3**	**Day 5**	**Day 10**	**Day 15**	**Day 20**	**Day 25**	**Day 30**	**Day 35**
	6.83	4.17	2.50	2.67	≤ 1.50	≤ 1.50	≤ 1.50	≤ 1.50	≤ 1.50
**Spruce**	5.83	≤ 3.67	≤ 2.00	≤ 1.50	≤ 1.50	≤ 1.50	≤ 1.50	≤ 1.50	≤ 1.50
	5.83	4.50	≤ 1.50	≤ 1.50	≤ 1.50	≤ 1.50	≤ 1.50	≤ 1.50	≤ 1.50
	4.33	≤ 3.33	≤ 1.50	≤ 1.50	≤ 1.50	≤ 1.50	≤ 1.50	≤ 1.50	≤ 1.50
**Pine**	5.00	≤ 2.50	≤ 1.50	≤ 1.50	≤ 1.50	≤ 1.50	≤ 1.50	≤ 1.50	≤ 1.50
	4.67	≤ 2.50	≤ 1.50	≤ 1.50	≤ 1.50	≤ 1.50	≤ 1.50	≤ 1.50	≤ 1.50
	8.00	6.33	4.67	≤ 1.67	3.83	3.00	≤ 1.83	≤ 1.50	≤ 1.50
**Poplar**	6.83	5.00	3.83	≤ 1.67	≤ 1.50	≤ 1.50	≤ 1.50	≤ 1.50	≤ 1.50
	7.17	5.33	4.83	3.83	3.17	≤ 2.33	≤ 1.50	≤ 1.50	≤ 1.50
	5.67	4.00	≤ 3.17	4.33	3.00	≤ 1.50	≤ 1.50	≤ 1.50	≤ 1.50
**Beech**	5.67	≤ 3.67	≤ 3.17	≤ 2.33	≤ 1.50	≤ 1.50	≤ 1.50	≤ 1.50	≤ 1.50
	5.67	4.00	4.00	≤ 2.33	≤ 1.83	≤ 2.33	≤ 1.50	≤ 1.50	≤ 1.50
	6.83	4.67	≤ 3.50	≤ 2.00	≤ 1.50	≤ 1.50	≤ 1.50	≤ 1.50	≤ 1.50
**Douglas fir**	6.67	4.33	≤ 3.00	≤ 2.00	≤ 1.50	≤ 1.50	≤ 1.50	≤ 1.50	≤ 1.50
	6.67	4.50	≤ 2.50	≤ 1.50	≤ 1.50	≤ 1.50	≤ 1.50	≤ 1.50	≤ 1.50
	8.00	6.17	6.17	5.33	4.00	≤ 2.33	3.00	≤ 2.00	≤ 1.67
**Steel**	7.83	7.00	6.33	5.67	4.50	2.83	2.83	3.00	≤ 2.00
	8.00	6.33	6.83	5.17	3.83	3.83	≤ 2.50	2.83	≤ 1.67
	7.50	7.50	7.33	7.33	7.50	7.33	7.67	7.17	7.00
**Steel***	8.00	7.50	7.33	7.50	7.33	7.33	7.33	7.17	7.00
	7.50	7.67	7.67	7.50	7.50	7.50	7.67	7.67	7.17

* completely dried in a desiccator and then stored airtightly sealed

**Initial titre[1]**	8.50	9.00	9.00

[1] Initial titre in suspension on the day of infection; virus used for all carriers
